# Target Trial Emulations of GLP‐1 and Dual GLP‐1/GIP Agonists to Reduce Major Adverse Liver Outcomes in Type 2 Diabetes

**DOI:** 10.1111/liv.70367

**Published:** 2025-09-22

**Authors:** Alex E. Henney, David R. Riley, Matthew Anson, Shazli Azmi, Uazman Alam, Daniel J. Cuthbertson

**Affiliations:** ^1^ Department of Cardiovascular & Metabolic Medicine University of Liverpool Liverpool UK; ^2^ Metabolism & Nutrition Research Group Liverpool University Hospitals NHS Foundation Trust Liverpool UK; ^3^ Liverpool Centre for Cardiovascular Sciences University of Liverpool and Liverpool University Hospitals NHS Foundation Trust Liverpool UK; ^4^ Division of Cardiovascular Sciences University of Manchester Manchester UK; ^5^ Centre for Musculoskeletal Research, Faculty of Biology Medicine and Health The University of Manchester Manchester UK

## Abstract

**Background:**

Clinical trials suggest GLP‐1 receptor agonists (RAs) and dual glucagon‐like peptide‐1 (GLP‐1)/glucose‐dependent insulinotropic polypeptide (GIP) RAs improve metabolic dysfunction associated with steatohepatitis (MASH) in patients with metabolic dysfunction‐associated steatotic liver disease (MASLD). We aimed to compare the estimate of the relative effect of tirzepatide, semaglutide, and liraglutide in reducing the risk of major adverse liver outcomes (MALOs) in patients with type 2 diabetes (T2D).

**Design, Setting and Participants:**

We emulated target trials based on a real‐world network of electronic health records (EHRs) from over 150 million patients. Three target trials were emulated, among eligible patients with T2D who had no prior MALO diagnosis, by comparing therapy involving tirzepatide, semaglutide, and liraglutide versus DPP4 inhibitor (DPP4i) therapy. We identified the first‐ever diagnosis of MALO occurring within a 2‐year follow‐up period and compared across the treatment groups using Kaplan–Meier survival analyses. Cohorts underwent propensity score matching 1:1 for confounders. We performed sensitivity analyses relating to geographical location, combination with metformin, and by treatment adherence. We also performed head‐to‐head analyses of the incretin‐based therapies.

**Results:**

After matching, we identified three target trials comprised of 10 165, 56 702, and 8 301 patients treated with tirzepatide, semaglutide, and liraglutide, respectively (1:1 with reference patients) for a 2‐year period. Tirzepatide (HR 0.53 [95% CI 0.40, 0.71]) and semaglutide (HR 0.81 [0.72, 0.90]) were associated with a significant reduction in the risk of incident MALO compared with DPP4i, whereas liraglutide was not (HR 1.04 [95% CI 0.79, 1.36]). In head‐to‐head comparisons, tirzepatide was associated with a significantly lower risk of incident MALO compared with liraglutide (HR 0.56 [95% CI 0.39, 0.79]), but not semaglutide (HR 0.83 [95% CI 0.63, 1.09]). Semaglutide was not associated with a reduced risk compared with liraglutide (HR 0.77 [95% CI 0.57, 1.05]).

**Conclusion:**

Treatment with tirzepatide and, to a lesser extent, semaglutide, in patients with T2D, was associated with a lower incidence of MALO compared with DPP4i after 2 years; largely driven by a reduction in the rates of compensated and decompensated cirrhosis. A reduction in MALO was not demonstrated with the use of liraglutide. These findings highlight a comparative benefit of tirzepatide (and semaglutide) versus DPP4i and should prompt more robust, longer‐term randomised controlled studies to evaluate their role in preventing MALO in this increasingly prevalent patient population with co‐existing T2D and MASLD.

**Summary**

- In this study of real‐world data, we find that two drugs, semaglutide (a GLP‐1 receptor agonist) and tirzepatide (a dual GLP‐1/GIP receptor agonist), reduced the risk of major liver problems in people with type 2 diabetes by 28% and 39%, respectively, compared to another common diabetes treatment.
- Liraglutide, another similar drug, did not show this same benefit. These findings suggest that tirzepatide and semaglutide could be promising treatments for patients with type 2 diabetes and liver issues, but more research is needed to confirm these results.

## 1 Introduction

Metabolic dysfunction‐associated steatotic liver disease (MASLD), the most common chronic liver disease globally [1], represents a histopathological spectrum ranging from simple steatosis (most commonly) through to metabolic dysfunction‐associated steatohepatitis (MASH), fibrosis, and cirrhosis. Although progression to cirrhosis and related sequelae is typically slow and occurs only in a subset, its epidemiological significance relates to the high population prevalence of MASLD. The risk of MALO is highly associated with the stage of fibrosis at diagnosis of MASLD; the 20‐year cumulative incidence was 3% for patients with F0 fibrosis but 35% for patients with F3 fibrosis (vs. 2% for the reference population) [2], accelerated by associated comorbid disease, including obesity, metabolic syndrome (MetS) traits, and type 2 diabetes (T2D) [3].

Recent management of T2D, obesity and the MetS has been transformed by such (second‐generation) glucagon‐like peptide‐1 receptor agonists (GLP‐1 RAs) as liraglutide and semaglutide, and, more recently, dual agonists of GLP‐1 and glucose‐dependent insulinotropic polypeptide (GIP) receptors, such as tirzepatide. The myriad clinical applications of these drugs include glucose lowering [4, 5, 6, 7, 8], weight loss, cardiorenal protection [9, 10, 11, 12], treatment of MASLD [13] and obstructive sleep apnoea (OSA) [14]. Specifically, in randomised controlled clinical trial data, treatment with both tirzepatide and semaglutide for 52 weeks is more effective than placebo in the resolution of MASH without worsening of fibrosis [15]. Moreover, two real‐world evidence studies have evaluated the impact of GLP‐1 RAs, but not tirzepatide, on MALO and found heterogeneous results [16, 17].

Thus, to date, real‐world evidence relating to the impact of tirzepatide on liver‐related outcomes is absent, whilst findings of previous real‐world evidence assessing semaglutide on the impact of reducing MALO risk in patients with MASLD may be influenced by residual bias relating to underlying alcohol consumption or liver disease [18] (without adjustment for baseline differences in liver biochemistry, relating to underlying alcohol consumption or liver inflammation/fibrosis) [18]. The potential public health implications of a potent drug to reverse the adverse histopathological features of MASLD, simultaneously improving cardiometabolic health, are enormous and such a therapeutic approach could, for the first time, reduce the rising tide of liver‐related mortality, which has thus far proved difficult to address [19]. Therefore, the aims of this study were to compare the effectiveness of tirzepatide, semaglutide, and liraglutide in reducing incident MALO in patients with type 2 diabetes.

## 2 Methods

### 2.1 Specification of the Target Trials

#### 2.1.1 Study Overview

We compared the new use of tirzepatide, semaglutide, and liraglutide with that of DPP4i on the time to a first‐time diagnosis of MALO using a target trial emulation framework. We selected DPP‐4 inhibitors (DPP4i) as the active comparator because they are widely used as second‐line agents in similar clinical contexts to tirzepatide and therefore help mitigate confounding by indication, healthcare contact, and disease severity. For example, the National Institute for Care Excellence 2025 guidelines have DPP4i and GLP‐1 RAs as second‐line treatment options behind metformin and SGLT2is [20]. Importantly, there is no consistent trial‐level evidence that DPP4i has any major impact on any disease outcomes, and significantly, this includes major adverse cardiovascular events (MACE), in contrast to many other classes of glucose‐lowering therapies. We would also point out its neutral effect on body weight and its modest impact on glycaemic control. There have been no published studies to suggest DPP4i influence major adverse liver outcomes (MALO). Taken together, considering its mechanism, mode of action, neutral impact on body weight, and modest glycaemic impact, we feel it provides the best clinically relevant active comparator to study MALO. We also note that other antidiabetic classes (e.g., SGLT2i) have accumulating evidence of liver benefit, which would have biased comparisons away from the null [21]. Supporting Information lists key protocol components. To enhance clarity, we explicitly separate the specification of the idealised target trial from its emulation in the TriNetX platform [22]. Table S1 summarises the key protocol components of the hypothetical pragmatic randomised clinical trial and the corresponding emulation strategies applied in our real‐world data analysis. The target trials are specified as follows:

### 2.2 Eligibility Criteria

*Inclusion criteria* for all target trials included patients with T2D who had medical encounters with a Health Care Organisation (HCO) between May 2022 and November 2023, were prescribed one of tirzepatide, semaglutide, liraglutide, or DPP4i during this time window, and were diagnosed with at least one co‐morbid cardiometabolic condition (obesity, hypertension, dyslipidaemia, or known to have cardiovascular disease (heart disease or stroke); all defined according to ICD‐10 codes) that would render them eligible for GLP‐1 (±GIP) RA prescription.

*Exclusions* included a diagnosis of type 1 diabetes, history of MALO or chronic liver disease prior to the index event, co‐prescription of any of the treatment or reference medications, contraindications and limited use information for tirzepatide, semaglutide, liraglutide or dulaglutide (history of pancreatitis, thyroid cancer, gallstones, gastroparesis), and patients cannot have been initiated on any other glucose‐lowering therapy within the past 6 months (“new user”).

*Baseline HbA*_*1c*_ Although some GLP‐1 RA trials mandate patients have a baseline HbA_1c_ between 7% and 10% on enrolment, we opted not to apply this inclusion criteria in the current study. We justify this because trials will often apply an upper HbA_1c_ limit of 10% for safety reasons, with insulin introduced as escalation therapy if HbA_1c_ remains very high or the patient is severely symptomatic on the trial drug. However, despite emulating a target trial, this aspect of glycaemic control is not a concern using real world data.

### 2.3 Treatment Strategies

In each of the three target trials, treatment strategies were either one of initiation of tirzepatide, semaglutide, or liraglutide at baseline (index event) or the initiation of DPP4i at baseline (index event), but not both. Indeed, patients could not have been co‐prescribed any of the drugs evaluated at any point in the electronic health record history. For all treatment strategies, initiation of use is defined as the first prescription for the drug, consistent with an intention‐to‐treat design. The treatment strategy is assigned at baseline, regardless of medication use adherence, medication switch, or add‐on. The index event followed an active comparator, new user design where the analysis was of new starters of each drug, 1 day after drug initiation. Patients were followed up for 2 years. The active comparator new user design offers results that are more directly applicable to clinical practice because the reference drug (DPP4i) is an alternative treatment option enhancing the generalisability of our findings to broader patient populations.

### 2.4 Study Outcomes

**FIGURE 1:**
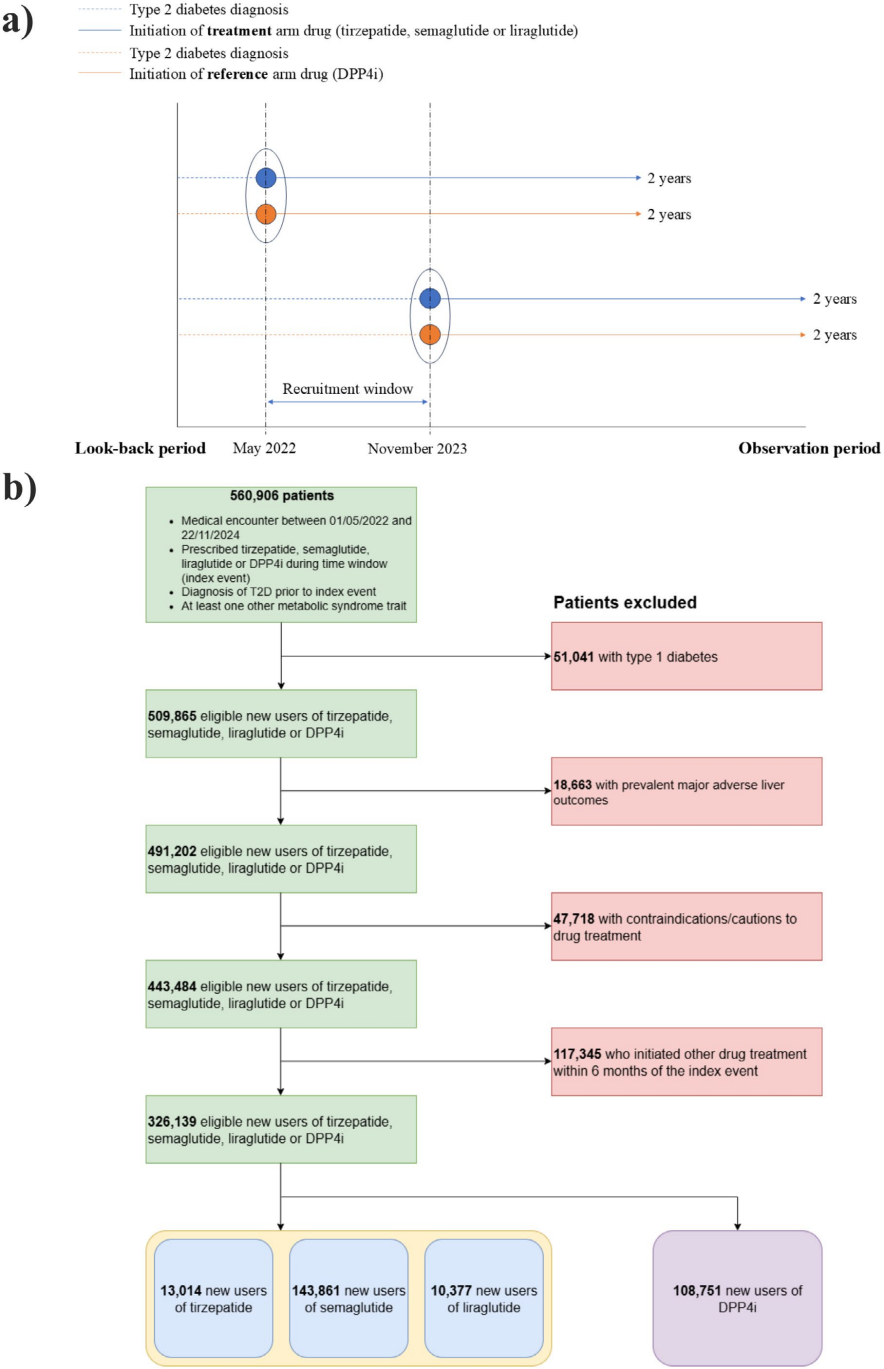
(a) Timeline of patient recruitment to the target trials. Patients were followed up for a maximum of 2 years from the index event (mean follow‐up time was 425 and 475 days in the tirzepatide versus DPP4i analysis, 494 and 490 days in the semaglutide vs. DPP4i analysis and 526 and 565 days in the liraglutide vs. DPP4i analysis), and (b) CONSORT diagram demonstrating the design of the three target trials comparing tirzepatide, semaglutide, liraglutide and dulaglutide to DPP4i.

*Primary outcome* was a first‐time diagnosis of (incident) MALO documented in patient electronic health records (EHRs). MALO was defined as a composite endpoint comprised of compensated or decompensated (bleeding varices, ascites, hepatorenal syndrome, hepatic encephalopathy), cirrhosis, chronic liver failure, hepatocellular carcinoma, or liver transplant. *Secondary outcomes* were the individual MALO endpoints. *Follow‐up*: Each eligible patient was followed from the index event until the occurrence of the outcome, death, loss to follow‐up, or 2 years after the index event, whichever occurred first. A timeline is presented in Figure 1a.

#### 2.4.1 Analysis Approach

The causal estimates of interest represent the intention‐to‐treat effect of being assigned to the treatment strategies. Cumulative incidences were estimated using the Kaplan–Meier survival analysis in patients who were propensity‐score matched (1:1 using nearest‐neighbour greedy matching with a calliper of 0.25 times the standard deviation) for baseline covariates. Hazard ratios (HRs) and 95% CIs were calculated. All models are adjusted for confounders at baseline by propensity‐score‐matching baseline covariates. There is a Cox regression running in parallel with the Kaplan–Meier survival analysis to calculate the hazard ratio. The difference is that the ‘Compare Outcomes’ TriNetX model is looking at the effect of just the index event on the outcome. This model in effect assumes all other aspects of the cohort are equal, which is why we propensity score match for possible confounders. While the user interface may not explicitly display a stratified Cox model or separate adjustment for the matched pairs, the PSM is performed prior to outcome comparison to balance baseline covariates between groups. This design ensures that the estimated hazard ratio compares well‐balanced cohorts, effectively controlling for confounding through matching. Unlike traditional Cox regression models which adjust for multiple covariates simultaneously, the TriNetX approach estimates the hazard ratio for the index event under the assumption that matched cohorts are comparable on measured confounders.

### 2.5 Emulation of the Target Trials

#### 2.5.1 Study Design

We explicitly emulated the target trials described previously using data and built‐in analytic functions on the TriNetX Analytics platform. TriNetX (LLC, Cambridge, MA, USA) is a global federated health research network with access to both inpatient and outpatient electronic medical records from health care organisations internationally; largely secondary and tertiary care providers in North America and Western Europe. This analysis was conducted on the Global Collaborative Network, which contains data from over 150 million patients, across over 140 HCOs, with access to diagnoses, procedures, medications, laboratory values, and genomic information worldwide. Data were collected in November 2024. The built‐in analytics within the TriNetX Analytic platform analysed patient‐level data; however, only population‐level results are reported to users. TriNetX data are HIPAA (Health Insurance Portability and Accountability Act) de‐identified, and access to protected health information is not allowed. Therefore, there is no risk for protected health information disclosure, and Institutional Review Board review was not required. Further details on the network have been described elsewhere [23].

Each component of the target trial was emulated using EHRs from the TriNetX Analytics platform. Patients were classified into drug groups—treatment arm (tirzepatide, semaglutide, liraglutide) or reference arm (DPP4i)—based on the first prescription in the study period (May 2022 to November 2023), which was the baseline or index event. The study period was chosen because tirzepatide was approved by the FDA to treat T2D in May 2022 and approved for weight loss in November 2023. Eligibility criteria and 40 baseline covariates were evaluated at baseline. This included a look‐back period set to “anytime” within the TriNetX Analytical platform; this is capped at a maximum of 20 years, and therefore the earliest date looked back to was 2004. The treatment and reference arms were separately propensity‐score matched for covariates at the baseline to emulate randomisation. After propensity‐score matching, all groups must have been considered well balanced using a standardised mean difference of < 0.1.

#### 2.5.2 Propensity Score Matching

Cohorts were propensity score matched (PSM), in a 1:1 ratio, for (i) sociodemographic variables: age, sex, ethnicity, smoking, alcohol‐use disorder (AUD) (AUD was included as a baseline covariate in propensity score matching. However, systematic data on alcohol consumption were not available within TriNetX, and therefore patients with moderate alcohol use and thus potential MetALD could not be reliably identified or excluded), socioeconomic status (problems relating to education and literacy, employment, housing, and psychosocial circumstances), (ii) comorbidities: cardiovascular disease (ischaemic heart disease (IHD), peripheral vascular disease (PVD), heart failure, cerebrovascular accident (CVA), hypertension and dyslipidaemia), (iii) anthropometrics: body mass index (BMI), and systolic and diastolic blood pressure, (iv) biochemistry: glomerular filtration rate (GFR), HbA1c, liver enzymes (alanine aminotransferase (ALT), aspartate aminotransferase (AST), and gamma glutamyl transferase (GGT)), clotting (prothrombin time (PT) and activated partial thromboplastin time (APTT), platelets, albumin and triglycerides), and (v) medication (other blood glucose‐lowering therapies: insulin, metformin, sulfonylureas, sodium‐glucose cotransporter‐2 inhibitors, thiazolidinediones, and other GLP‐1 RAs), corticosteroids, diuretics, and aspirin. All biochemical and anthropometric variables used in the cohort creation (i.e., HbA1c and BMI) must have been the most recent recorded value prior to the index event; however, we cannot state the exact duration for each patient as we do not have individual level data. Definitions for all PSM covariates are presented in Table S2.

#### 2.5.3 Statistical Analysis

Statistical analysis was performed in situ within TriNetX. TriNetX uses the R Survival package v3.2‐3. Additionally, for sensitivity analysis, we performed head‐to‐head analyses of the incretin‐based therapies (tirzepatide vs. (i) semaglutide, and (ii) liraglutide), replicated analysis in patients from the US collaborative network only to capture a more refined geographical location, mandated that treatment and reference arm drugs must have been add‐on therapy to metformin, and that treatment was adhered to for a minimum of 6 months. In the adherence sensitivity analysis, patients in both treatment and comparator arms were required to have evidence of persistence on the initiated therapy for at least 6 months. Follow‐up time remained anchored at the index date (day after drug initiation), such that the adherence requirement functioned as an inclusion criterion rather than altering the origin of follow‐up, thereby avoiding immortal time bias. Finally, we calculated E‐values, representing the minimum strength of association on the HR scale that an unmeasured confounder would need to have with both the exposure (treatment arm) and the outcome, conditional on the measured confounders, to explain away the observed association; HR + √[HR × (HR − 1)] [24]. The Strengthening the Reporting of Observational Studies in Epidemiology (STROBE) guidelines were followed in the reporting of this cohort study [25].

## 3 Results

### 3.1 Study Population

A CONSORT diagram (Figure 1) demonstrates the cohort composition and reasons for exclusions. The study cohort included 326 139 new users of the treatment arm drugs reduced to three treatment arms of new users of tirzepatide (13 014), semaglutide (143 861), liraglutide (10 377) and of reference arm, DPP4i (108 571), after excluding patients with co‐prescription of any of the study medication (Table 1).

**TABLE 1:** Baseline covariates of individuals in each treatment group.

Characteristic	Before propensity score matching			After propensity score matching		
	Treatment	Reference	SMD	Treatment	Reference	SMD
**Tirzepatide vs. DPP4i**						
*Demographics*						
Numbers (*n*)	13 014	108 751		10 165	10 165	
Age (years)	56 ± 12	68 ± 12	1.05	58 ± 11	58 ± 13	< 0.01
Sex, female (%)	59.1	48.0	0.22	56.8	55.7	0.02
Ethnicity, white (%)	70.8	35.9	0.75	68.8	70.3	0.03
Socioeconomic hazards (%)	3.0	2.5	0.03	3.1	3.2	< 0.01
Nicotine dependence (%)	11.1	8.8	0.08	11.4	12.2	0.03
Alcohol‐use disorder (%)	1.9	1.8	0.01	2.1	2.2	0.01
*Biochemistry (data completeness (%))*						
HbA1c (%)	7.4 ± 1.7	7.4 ± 1.5	0.02	7.4 ± 1.7	7.6 ± 1.8	0.10
ALT (U/L)	31 ± 24	25 ± 26	0.27	30 ± 21	29 ± 21	0.09
AST (U/L)	26 ± 17	24 ± 28	0.07	25 ± 16	25 ± 16	0.05
Albumin (g/dL)	4 ± < 1	4 ± 1	0.12	4 ± < 1	4 ± < 1	0.03
Platelets (×10^9^/L)	265 ± 75	241 ± 79	0.32	261 ± 74	258 ± 78	0.04
Prothrombin time (s)	13 ± 4	13 ± 4	0.08	13 ± 4	13 ± 4	0.07
Activated partial thromboplastin time (s)	31 ± 11	31 ± 10	0.01	32 ± 11	31 ± 12	< 0.01
eGFR (mL/min/1.73 m^2^)	81 ± 25	69 ± 30	0.45	80 ± 25	80 ± 28	< 0.01
*Anthropometrics (data completeness (%))*						
Body mass index (kg/m^2^)	38.3 ± 8.0	29.7 ± 6.7		37.3 ± 7.7	35.8 ± 7.6	
< 25			0.49			0.02
25–30			0.34			0.02
30–35			0.19			0.04
35–40			0.55			0.03
40–45			0.57			0.02
45–50			0.47			0.01
50–55			0.35			0.01
55–60			0.25			0.01
60–65			0.15			< 0.01
65–70			0.11			0.01
> 70			0.04			0.02
*Comorbidity (%)*						
Ischaemic heart disease	15.6	28.5	0.32	17.4	16.9	0.01
Cerebrovascular accident	6.3	16.2	0.32	7.3	7.3	< 0.01
Peripheral vascular disease	3.9	6.2	0.11	4.3	4.6	0.01
Hypertension	70.1	78.3	0.19	72.2	72.9	0.01
Dyslipidaemia	68.0	78.3	0.18	70.4	71.2	0.02
*Liver disease*						
MASLD	11.6	5.6	0.22	10.7	10.8	< 0.01
MASH	1.5	0.7	0.08	1.3	1.4	< 0.01
Viral hepatitis	0.7	2.8	0.17	0.7	0.8	0.01
Haemochromatosis	0.2	0.1	0.01	0.2	0.2	0.01
Wilson's disease	0.1	< 0.1	0.03	0.1	< 0.1	0.04
*Concomitant medication*						
Metformin	61.7	73.9	0.26	63.1	63.8	0.01
Insulin	33.6	38.7	0.11	33.8	34.8	0.02
Glipizide	9.9	16.8	0.20	11.2	12.0	0.02
Glimepiride	7.3	21.9	0.42	8.2	8.0	0.01
Glyburide	2.4	4.9	0.01	2.6	2.5	0.01
Pioglitazone	4.9	12.2	0.26	5.3	5.5	0.01
Empagliflozin	15.6	13.0	0.08	15.3	15.4	0.01
Dapagliflozin	7.5	7.4	< 0.01	7.4	7.6	0.01
Canagliflozin	3.2	3.5	0.01	3.4	3.6	0.01
Dulaglutide	26.0	3.2	0.68	18.	16.8	0.03
Exenatide	2.7	0.7	0.16	2.3	2.3	< 0.01
Corticosteroids	62.6	50.9	0.24	60.8	61.6	0.02
Aspirin	27.9	42.3	0.30	30.1	30.7	0.01
Diuretics	43.6	43.6	< 0.01	43.9	44.6	0.01
**Semaglutide vs. DPP4i**						
*Demographics*						
Numbers (*n*)	143 861	108 751		56 702	56 702	
Age (years)	58 ± 12	68 ± 12	0.85	64 ± 11	64 ± 12	0.02
Sex, female (%)	54.5	47.4	0.14	48.5	48.4	< 0.01
Ethnicity, white (%)	61.8	35.5	0.55	53.5	54.4	0.02
Socioeconomic hazards (%)	4.3	2.6	0.09	3.6	3.5	< 0.01
Nicotine dependence (%)	13.4	8.9	0.14	12.7	12.7	< 0.01
Alcohol‐use disorder (%)	2.5	1.8	0.04	1.7	1.6	0.01
*Biochemistry (data completeness (%))*						
HbA1c (%)	7.5 ± 1.8	7.4 ± 1.5	0.06	7.6 ± 1.7	7.6 ± 1.6	0.04
ALT (U/L)	29 ± 26	25 ± 26	0.19	27 ± 20	26 ± 27	0.05
AST (U/L)	25 ± 16	24 ± 28	0.03	24 ± 14	24 ± 24	< 0.01
Platelets (×10^9^/L)	263 ± 75	241 ± 79	0.29	252 ± 73	248 ± 78	0.06
Albumin (g/dL)	4 ± < 1	4 ± 1	0.23	4 ± < 1	4 ± 1	0.14
Prothrombin time (s)	13 ± 4	13 ± 4	0.10	13 ± 4	13 ± 5	0.02
Activated partial thromboplastin time (s)	31 ± 11	31 ± 10	0.01	32 ± 11	32 ± 11	< 0.01
eGFR (mL/min/1.73 m^2^)	80 ± 26	69 ± 30	0.40	74 ± 26	73 ± 29	0.05
*Anthropometrics (data completeness (%))*						
Body mass index (kg/m^2^)	36.6 ± 8.1	29.7 ± 6.7		33.3 ± 7.2	31.9 ± 6.8	
< 25			0.34			< 0.01
25–30			0.14			0.02
30–35			0.32			< 0.01
35–40			0.58			0.01
40–45			0.55			0.03
45–50			0.44			0.04
50–55			0.33			0.03
55–60			0.23			0.03
60–65			0.15			0.02
65–70			0.11			0.01
> 70			0.06			0.01
*Comorbidity (%)*						
Ischaemic heart disease	19.7	28.6	0.21	25.4	25.4	< 0.01
Cerebrovascular accident	8.5	16.3	0.24	12.4	12.3	< 0.01
Peripheral vascular disease	5.0	6.2	0.05	6.5	6.6	< 0.01
Hypertension	76.5	78.4	0.05	79.7	79.8	< 0.01
Dyslipidaemia	75.7	76.3	0.02	78.8	78.6	< 0.01
*Liver disease (%)*						
MASLD	12.7	5.7	0.25	8.6	8.4	0.01
MASH	1.6	0.7	0.08	1.0	1.0	< 0.01
Viral hepatitis	1.1	2.8	0.12	1.7	1.6	0.01
Haemochromatosis	0.3	0.1	0.03	0.2	0.2	< 0.01
Wilson's disease	< 0.1	< 0.1	< 0.1	< 0.1	< 0.1	< 0.1
*Concomitant medication*						
Metformin	69.6	73.8	0.09	72.3	72.0	0.01
Insulin	38.1	38.7	0.01	41.4	41.2	< 0.01
Glipizide	15.9	16.8	0.02	19.6	19.7	< 0.01
Glimepiride	10.9	21.9	0.30	15.2	14.8	0.01
Glyburide	3.2	4.9	0.09	4.2	4.2	< 0.01
Pioglitazone	6.8	12.1	0.18	9.4	9.2	0.01
Empagliflozin	18.2	13.1	0.14	17.3	17.1	0.01
Dapagliflozin	8.3	7.4	0.03	7.9	7.8	< 0.01
Canagliflozin	4.3	3.5	0.04	4.5	4.5	< 0.01
Dulaglutide	12.2	3.2	0.34	6.5	5.7	0.03
Exenatide	2.7	0.7	0.16	1.4	1.2	0.02
Corticosteroids	65.4	51.2	0.29	59.7	59.9	< 0.01
Aspirin	34.5	42.3	0.16	40.9	41.0	< 0.01
Diuretics	47.5	43.6	0.08	47.3	47.7	0.01
**Liraglutide vs. DPP4i**						
*Demographics*						
Numbers (*n*)	10 377	108 571		8301	8301	
Age (years)	57 ± 17	68 ± 12	0.78	59 ± 15	59 ± 14	0.05
Sex, female (%)	56.8	47.4	0.19	56.0	55.2	0.02
Ethnicity, white (%)	56.5	35.5	0.43	56.9	59.8	0.06
Socioeconomic hazards (%)	6.5	2.6	0.19	6.0	6.1	< 0.01
Nicotine dependence (%)	15.9	8.9	0.21	16.0	17.3	0.04
Alcohol‐use disorder (%)	3.0	1.8	0.08	3.1	3.4	0.02
*Biochemistry (data completeness (%))*						
HbA1c (%)	7.6 ± 2.1	7.4 ± 1.5	0.10	7.6 ± 2.0	7.8 ± 1.9	0.12
ALT (U/L)	28 ± 25	25 ± 26	0.13	27 ± 24	28 ± 51	0.01
AST (U/L)	25 ± 21	24 ± 28	0.02	24 ± 20	25 ± 51	0.02
Platelets (×10^9^/L)	263 ± 78	241 ± 79	0.28	260 ± 77	256 ± 80	0.06
Albumin (g/dL)	4 ± < 1	4 ± 1	< 0.01	4 ± < 1	4 ± 1	0.02
Prothrombin time (s)	13 ± 4	13 ± 4	0.16	13 ± 4	13 ± 4	0.02
Activated partial thromboplastin time (s)	31 ± 11	31 ± 10	0.01	32 ± 11	31 ± 11	0.01
eGFR (mL/min/1.73 m^2^)	77 ± 30	69 ± 30	0.29	77 ± 29	77 ± 32	0.01
*Anthropometrics (data completeness (%))*						
Body mass index (kg/m^2^)	36.2 ± 8.6	29.7 ± 6.7		35.7 ± 8.3	34.8 ± 7.8	
< 25			0.30			0.01
25–30			0.10			0.02
30–35			0.30			0.03
35–40			0.50			0.02
40–45			0.53			0.01
45–50			0.44			0.01
50–55			0.34			0.02
55–60			0.25			0.02
60–65			0.18			0.02
65–70			0.12			0.01
> 70			0.07			0.01
*Comorbidity (%)*						
Ischaemic heart disease	22.9	28.6	0.13	23.7	24.1	0.01
Cerebrovascular accident	11.1	16.3	0.15	11.5	11.5	< 0.01
Peripheral vascular disease	5.7	6.2	0.02	6.9	7.1	0.01
Hypertension	75.9	78.4	0.06	77.3	77.9	0.02
Dyslipidaemia	72.3	76.3	0.09	73.3	74.8	0.03
*Liver disease (%)*						
MASLD	11.2	5.7	0.20	10.6	10.4	0.01
MASH	1.7	0.7	0.09	1.6	1.4	0.01
Viral hepatitis	1.6	2.8	0.08	1.7	1.8	0.01
Haemochromatosis	0.1	0.1	< 0.01	0.2	0.2	0.01
Wilson's disease	0.1	< 0.1	0.04	0.1	0	0.05
*Concomitant medication*						
Metformin	68.7	73.8	0.11	68.1	67.8	0.01
Insulin	57.4	38.7	0.38	56.9	58.6	0.04
Glipizide	16.3	16.9	0.01	16.9	17.6	0.02
Glimepiride	9.9	21.9	0.33	10.3	10.8	0.02
Glyburide	4.3	4.9	0.03	4.5	4.3	0.01
Pioglitazone	6.7	12.1	0.19	6.9	6.9	< 0.01
Empagliflozin	13.9	13.1	0.02	14.3	15.1	0.02
Dapagliflozin	5.9	7.4	0.06	6.1	5.9	0.01
Canagliflozin	4.6	3.5	0.06	4.8	4.9	0.01
Dulaglutide	9.3	3.2	0.27	9.0	9.4	0.01
Exenatide	4.1	0.7	0.23	3.6	3.4	0.01
Corticosteroids	62.5	51.2	0.23	62.3	63.1	0.02
Aspirin	39.7	42.3	0.05	41.0	42.7	0.03
Diuretics	49.0	43.6	0.11	50.2	51.3	0.02

#### 3.1.1 Target Trial 1: Tirzepatide Versus DPP4i

##### 3.1.1.1 Baseline Characteristics

A total of 121 585 patients were identified: 13 014 (10.7%) prescribed tirzepatide, and 108 571 (89.3%) prescribed DPP4is. Before matching, those in the tirzepatide arm were on average younger, more likely to be white females with a higher serum ALT concentration, platelet count, eGFR, and BMI, but less likely to be living with comorbid disease including IHD, CVA, PVD, or hypertension. Finally, they were less commonly co‐prescribed glucose‐lowering therapy and aspirin. After PSM, each cohort was deemed well matched. The total number of participants in each cohort was reduced to 10 165 (Table 1).

##### 3.1.1.2 Survival Analysis

**FIGURE 2:**
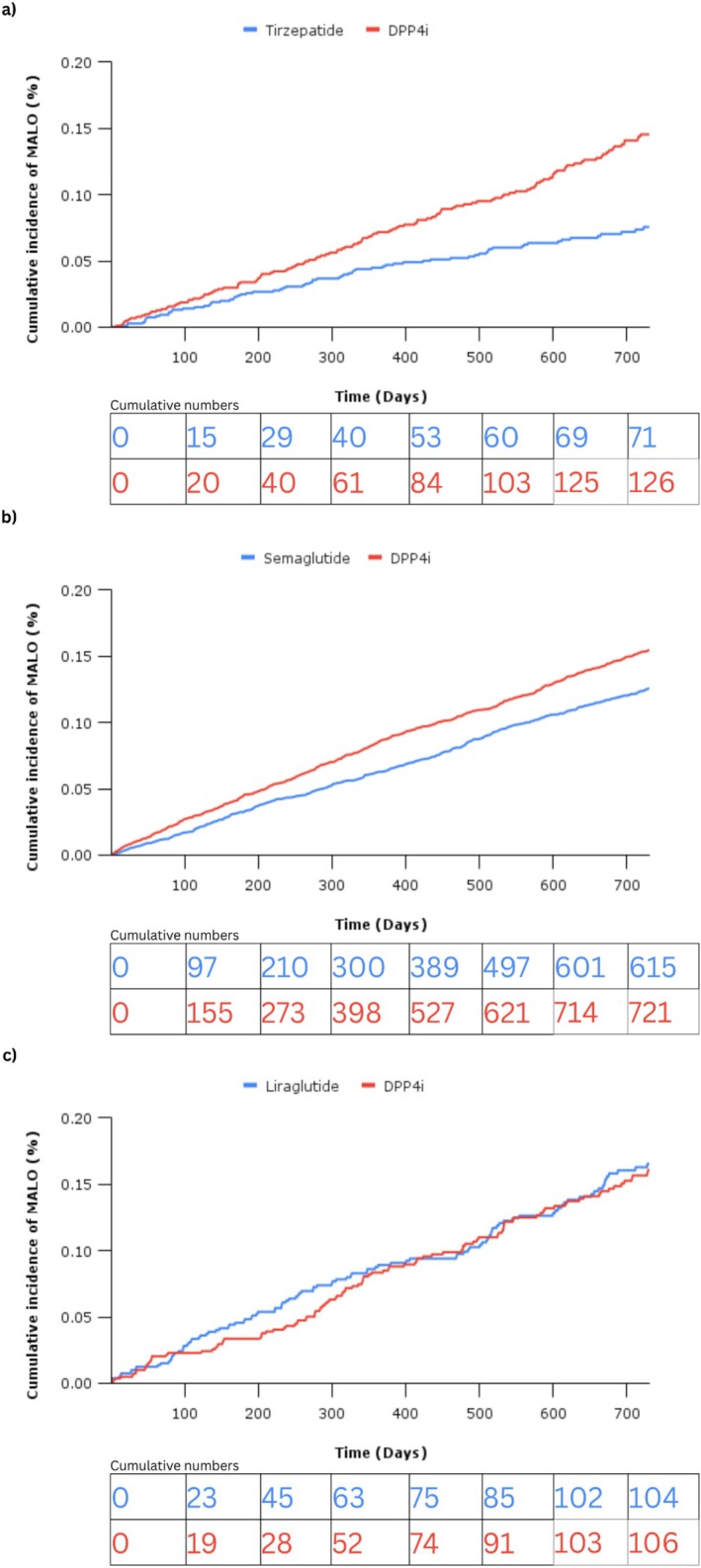
Cumulative incidence curves demonstrating the proportion of patients diagnosed with a major adverse liver outcome (MALO) during a maximum of 2 years of follow in patients treated with (a) tirzepatide, (b) semaglutide, and (c) liraglutide.

**FIGURE 3:**
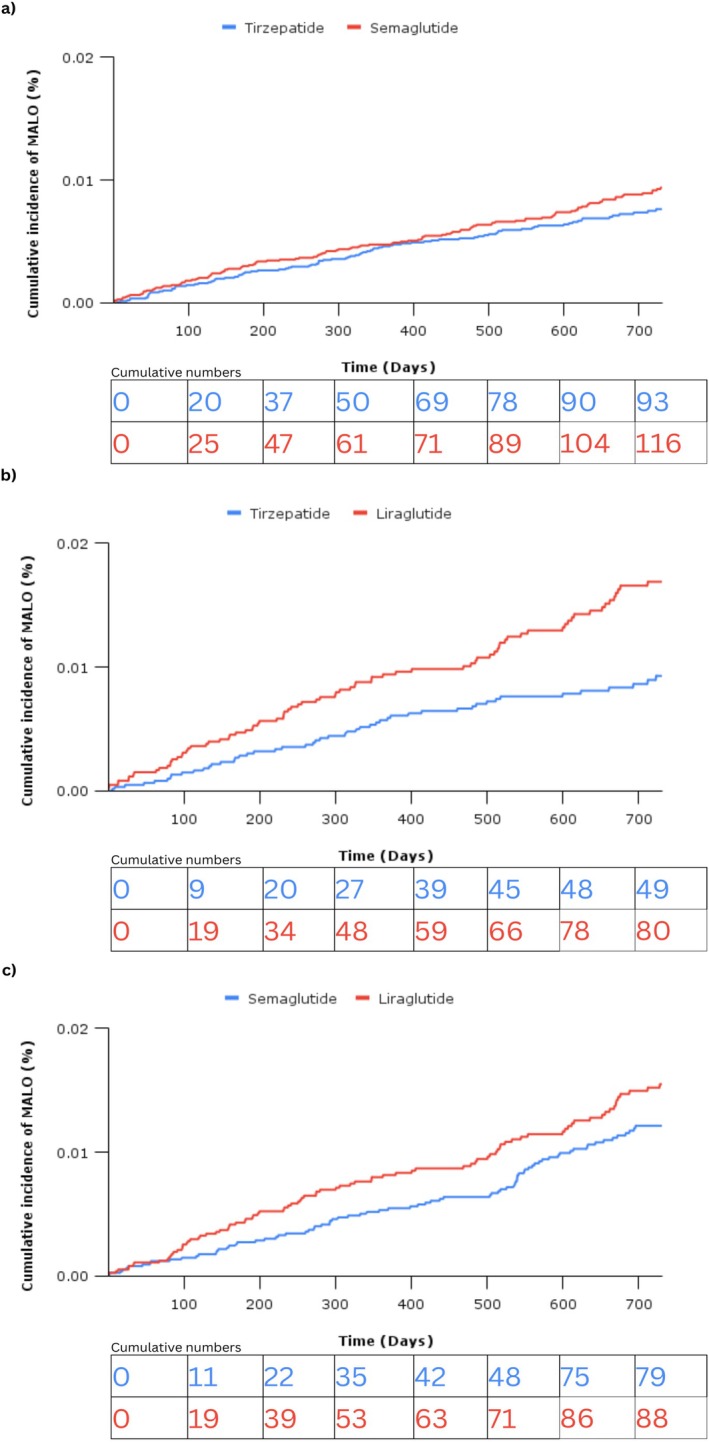
Cumulative incidence curves demonstrating the proportion of patients diagnosed with a major adverse liver outcome (MALO) during a maximum of 2 years of follow in patients treated with (a) tirzepatide compared to semaglutide, (b) tirzepatide compared to liraglutide, and (c) semaglutide compared to liraglutide.

Tirzepatide was associated with a reduced risk of incident MALO (HR 0.53 [95% CI 0.40, 0.71]) (Table 2). Survival curves are presented in Figure 2, with a forest plot in Figure 3. The incidence rate in the tirzepatide arm for the first diagnosis of MALO was 6.5 (vs. 11.6 in the DPP4i arm), per 1000 person‐years. Specifically, tirzepatide was associated with a reduced risk of cirrhosis (HR 0.57 [95% CI 0.38, 0.86]) and decompensated cirrhosis (HR 0.45 [95% CI 0.30, 0.67]), largely driven by ascites risk reduction (HR 0.43 [95% CI 0.28, 0.65]). The mean follow‐up time was 620 and 587 days in the tirzepatide and DPP4i arms, respectively.

**TABLE 2:** Major adverse liver outcomes as a composite measure with individual events in patients treated with tirzepatide, semaglutide, or liraglutide Versus a DPP‐4 inhibitor.

	Sample size	Outcome (*n*)	5‐year survival (%)	Hazard ratio [95% confidence interval]	Log‐rank test	*p*	*E*
**Tirzepatide vs. DPP4i**							
***Major adverse liver outcomes (composite)***							
Reference	10 843	126	98.5	Reference			
Tirzepatide	10 843	71	99.2	0.53 [0.40, 0.71]	18.5	< 0.01	
***Individual MALO components***							
*Cirrhosis*							
Reference	10 843	61	99.3	Reference			
Tirzepatide	10 843	37	99.6	0.57 [0.38, 0.86]	7.3	0.01	
*Decompensated cirrhosis*							
Reference	10 843	78	99.1	Reference			
Tirzepatide	10 843	37	99.6	0.45 [0.30, 0.67]	16.9	< 0.01	
*Hepatocellular carcinoma*							
Reference	10 843	< 10^a^	99.9	Reference			
Tirzepatide	10 843	< 10^a^	99.9	0.67 [0.26, 1.77]	0.65	0.42	1.00
*Liver transplant*							
Reference	10 843	0	100	Reference			
Tirzepatide	10 843	< 10^a^	< 100	NA	1.9	0.17	1.00
**Semaglutide vs. DPP4i**							
***Major adverse liver outcomes (composite)***							
Reference	56 702	721	99.5	Reference			
Semaglutide	56 702	615	98.7	0.81 [0.72, 0.90]	15.5	< 0.01	
***Individual MALO components***							
*Cirrhosis*							
Reference	56 702	337	99.3	Reference			
Semaglutide	56 702	342	99.3	0.96 [0.83, 1.12]	0.3	0.60	1.00
*Decompensated cirrhosis*							
Reference	56 702	441	99.0	Reference			
Semaglutide	56 702	283	99.4	0.61 [0.52, 0.70]	44.4	< 0.01	
*Hepatocellular carcinoma*							
Reference	56 702	81	99.8	Reference			
Semaglutide	56 702	70	99.9	0.82 [0.59, 1.12]	1.6	0.21	1.00
*Liver transplant*							
Reference	56 702	< 10^a^	> 99.9	Reference			
Semaglutide	56 702	22	> 99.9	2.6 [1.17, 5.91]	5.9	0.01	
**Liraglutide vs. DPP4i**							
***Major adverse liver outcomes (composite)***							
Reference	8301	106	98.4	Reference			
Liraglutide	8301	104	98.3	1.04 [0.79, 1.36]	0.1	0.79	1.00
***Individual MALO components***							
*Cirrhosis*							
Reference	8301	55	99.2	Reference			
Liraglutide	8301	64	99.0	1.23 [0.86, 1.77]	1.3	0.25	1.00
*Decompensated cirrhosis*							
Reference	8301	73	99.9	Reference			
Liraglutide	8301	54	99.1	0.78 [0.55, 1.12]	1.8	0.18	1.00
*Hepatocellular carcinoma*							
Reference	8301	< 10^a^	> 99.9	Reference			
Liraglutide	8301	14	99.8	4.83 [0.89, 16.82]	7.5	0.06	1.00
*Liver transplant*							
Reference	8301	< 10^a^	> 99.9	Reference			
Liraglutide	8301	< 10^a^	> 99.9	1.05 [0.07, 16.82]	< 0.01	0.97	1.00

*Note:* The survival analysis is performed using the true values but is blinded to the research team.

^a^ In TriNetX, any outcome with an event count < 10 is reported as < 10 rather than the true value in line with their confidentiality and data sharing agreement.

##### 3.1.1.3 Stratified Analysis

Tirzepatide was associated with a reduced risk of incident MALO when analysed in patients from the USA only (HR 0.57 [95% CI 0.42, 0.75]), used as adjunctive therapy to metformin (HR 0.57 [95% CI 0.39, 0.83]) and when treatment was adhered to for a minimum of 6 months (HR 0.57 [95% CI 0.40, 0.80]) (Table S3).

#### 3.1.2 Target Trial 2: Semaglutide Versus DPP4i

##### 3.1.2.1 Baseline Characteristics

A total of 252 432 patients were identified: 143 861 (57%) prescribed semaglutide and 108 571 (43%) prescribed DPP4i. Before matching, those in the semaglutide arm were on average younger and more likely to be white females who smoke. They had higher ALT, platelets, albumin, eGFR, and BMI, but were less likely to be living with prevalent comorbid disease (IHD or CVA). Finally, they were less likely to be co‐prescribed another glucose‐lowering therapy and aspirin. After PSM, each cohort was deemed well matched. The total number of participants in each cohort was reduced to 56 702 (Table 1).

##### 3.1.2.2 Survival Analysis

Semaglutide was associated with a reduced risk of incident MALO (HR 0.81 [95% CI 0.72, 0.90]) (Table 2). Survival curves are presented in Figure 2, with a forest plot in Figure 3. The incidence rate in the semaglutide arm for the first diagnosis of MALO was 10.8 (vs. 12.7 in the DPP4i arm), per 1000 person‐years. Specifically, semaglutide was associated with a reduced risk of decompensated cirrhosis (HR 0.61 [95% CI 0.52, 0.70]), which was largely the result of ascites (HR 0.60 [95% CI 0.51, 0.70]) and encephalopathy (HR 0.50 [95% CI 0.29, 0.87]) risk reduction. The mean follow‐up time was 627 and 592 days in the semaglutide and DPP4i arms, respectively.

##### 3.1.2.3 Stratified Analysis

Semaglutide was associated with a reduced risk of incident AUD when analysed in patients from the USA only (HR 0.74 [95% CI 0.66, 0.83]), when treatment was adhered to for a minimum of 6 months (HR 0.75 [95% CI 0.65, 0.86]), and when used as adjunctive therapy to metformin (HR 0.75 [95% CI 0.65, 0.86]) (Table S3).

#### 3.1.3 Target Trial 3: Liraglutide Versus DPP4i

##### 3.1.3.1 Baseline Characteristics

A total of 118 948 patients were identified: 10 377 (8.7%) prescribed liraglutide and 108 571 (91.3%) prescribed DPP4is. Before matching, the liraglutide arm was, on average, younger, more likely to be female, white, smokers and to have adverse socioeconomic status. They had a lower HbA1c but higher ALT, platelets, prothrombin time, eGFR, and BMI. They were more also less likely to be living with prevalent comorbid disease (IHD or CVA). After PSM, each cohort was deemed well matched. The total number of participants in each cohort was reduced to 8301 (Table 1).

##### 3.1.3.2 Survival Analysis

Liraglutide was not associated with a reduced risk of incident MALO (HR 1.04 [95% CI 0.79, 1.36]) (Table 2). Survival curves are presented in Figure 2, with a forest plot in Figure 3. However, liraglutide was associated with reduced risk of the individual ascites endpoint (HR 0.67 [95% CI 0.45, 0.99]). The incidence rate in the semaglutide arm for the first diagnosis of MALO was 12.5 (vs. 12.8 in the DPP4i arm), per 1000 person‐years. Liraglutide was not associated with a risk reduction in any secondary outcome. The mean follow‐up time was 550 and 581 days in the liraglutide and DPP4i arms, respectively.

##### 3.1.3.3 Stratified Analysis

Liraglutide was not associated with a reduced risk of incident MALO when analysed in patients from the USA only, when used as add‐on therapy to metformin or when treatment was adhered to for a minimum of 6 months (Table S3).

### 3.2 Head‐to‐Head Analysis

Tirzepatide was associated with a reduced risk of incident MALO compared to liraglutide (HR 0.56 [95% CI 0.39, 0.79]), but not semaglutide (HR 0.83 [95% CI 0.63, 1.09]). Semaglutide was not associated with a reduced risk of MALO when compared against liraglutide (HR 0.77 [95% CI 0.57, 1.05]) (Figure 3).

## 4 Discussion

In the first study to evaluate and compare the effect estimates of three incretin‐based receptor agonists (tirzepatide, semaglutide, and liraglutide) in reducing incident MALO in patients with T2D, we demonstrate that the use of tirzepatide was associated with a 47% reduction in the risk of incident MALO over 2 years, driven by risk reductions in cirrhosis and decompensated cirrhosis, particularly the development of ascites. Treatment with semaglutide was also associated with a significant, albeit more modest, 19% reduction in composite MALO risk. In contrast, liraglutide was not associated with a reduction in MALO, except for a signal of reduced ascites risk. In head‐to‐head comparisons, tirzepatide conferred a significantly lower risk of MALO than liraglutide, but its effect size was not significantly different from semaglutide.

Meta‐analysis of eight clinical trials in patients with T2D and MASLD demonstrated a reduction in MRI‐measured hepatic fat when treated with GLP‐1 RAs [26], whilst 24 weeks of semaglutide, specifically, improved liver fat and stiffness as assessed via transient elastography [27]. One semaglutide RCT, including 320 participants with MASH, reported that the proportion achieving MASH resolution without worsening fibrosis after 72 weeks was more than tripled in the arm receiving 0.4 mg semaglutide compared with placebo (59% vs. 17%) [28]; however, a second trial in 71 participants with MASLD‐related cirrhosis found that semaglutide at a higher dose of 2.4 mg did not significantly improve fibrosis or achievement of MASH resolution [13]. A single tirzepatide RCT found that, in 190 participants with MASH and moderate or severe fibrosis, tirzepatide was more effective than placebo in the resolution of MASH without worsening of fibrosis [15].

Several cohort studies have examined the impact of GLP‐1 receptor agonists on MALO in patients with liver disease and T2D; collectively presented by a 29% risk reduction in MALO during meta‐analysis [29]. A nationwide Swedish target trial reported a 49% lower 10‐year risk of MALO among GLP‐1 RA initiators with chronic liver disease and T2D, though estimates were imprecise (95% CI 0.50–1.32; *n* = 1026 vs. 15 633) [16]. Using MarketScan data, another study of MASLD cirrhosis with T2D (459 GLP‐1 RA users vs. 4837 non‐users) found lower risks of compensated cirrhosis (HR 0.64 [0.50–0.80]), hepatocellular carcinoma (HR 0.47 [0.22–0.98]), and liver transplantation (HR 0.51 [0.27–0.93]) [17]. Neither study assessed tirzepatide or compared individual GLP‐1 RAs, and both lacked an active comparator. More recently, a large Scandinavian cohort comparing GLP‐1 RAs with DPP‐4 inhibitors reported a lower risk of serious liver events (adjusted HR 0.85 [0.75–0.97]), driven by reduced cirrhosis, with no clear effect on hepatocellular carcinoma (HR 1.05 [0.80–1.39]) [30]. Collectively, these findings are concordant with our results, though our study provides agent‐specific data and includes tirzepatide, using an active comparator design.

In MASLD populations without mandatory T2D, a TriNetX study (*n* = 6243 per group after PSM) reported a 54% lower 7‐year risk of clinically significant portal hypertension events among GLP‐1 RA users versus non‐users, though the absence of an active comparator and possible unmeasured confounding limits interpretation [31].

The observed superiority of tirzepatide compared with semaglutide in reducing incident MALO may relate to its dual agonism of the GLP‐1 and GIP receptors. Beyond the established benefits of GLP‐1 receptor activation on weight loss, whole body insulin sensitivity, and hepatic steatosis, and possibly fibroinflammation, GIP receptor agonism has been shown to exert complementary metabolic effects, including enhanced lipid handling, adipose tissue remodelling, and improved insulin action. Experimental studies further suggest that dual GLP‐1/GIP receptor agonism may attenuate inflammatory signalling and fibrogenic pathways more effectively than GLP‐1 receptor agonism alone [31]. These synergistic mechanisms could plausibly account for the more pronounced reduction in MALO risk observed with tirzepatide compared to semaglutide in our study.

Our findings may carry important potential clinical implications. In patients with T2D, a population enriched with coexistent MASLD, tirzepatide and semaglutide are both associated with significant reductions in the risk of MALO. Tirzepatide also demonstrates greater efficacy than liraglutide in head‐to‐head analyses. These results suggest that, where clinically appropriate, tirzepatide or semaglutide may be preferable to liraglutide or DPP4 inhibitors in patients with T2D at risk of liver disease progression, particularly given the presence of MASLD with accompanying hepatic fibrosis. Given that MASLD, with and without fibrosis, is highly prevalent among individuals with T2D, the dual benefit of these therapies in improving both cardiometabolic and hepatic outcomes may inform treatment decisions and guideline development. However, while our data support the preferential use of tirzepatide and semaglutide in this context, confirmatory evidence from longer‐term randomised controlled trials will be essential before formal treatment recommendations can be established.

There are limitations that must be acknowledged. First, these are real‐world data, and comparisons are not randomised, nor controlled. Second, there is potential for a lack of data completeness resulting from data being extracted from EHRs of an administrative database. For example, data may not be recorded by the HCO, or other data recorded in free text, which we are unable to extrapolate. In addition, should participants move between HCOs, it is possible that some of their data may not be available to us as one or more of their HCOs may not form part of the global collaborative network. Third, information concerning dosage and rate of dose escalation of tirzepatide, semaglutide, and liraglutide was not available to us, and we were unable to comment on the dose‐dependent relationship of incretin‐based therapies on incident MALO. However, to navigate this challenge surrounding dosing, we refined our inclusion criteria for the respective drugs to the dates between which they were approved for T2D (lower drug dose) and obesity (higher drug dose). Fourth, our findings are limited by a short duration of follow‐up due to the novelty of tirzepatide as a treatment for T2D. This is an important consideration given that certain individual MALO endpoints (cirrhosis and HCC, for example) may take decades to present in patients with MASLD [32]. Fifth, as with any large database study, residual confounding remains possible. To help readers gauge the potential impact of unmeasured confounding, we calculated E‐values, which quantify the minimum strength of association that an unmeasured confounder would need to have with both the exposure and the outcome to fully explain away the observed associations [24]. Alcohol consumption is poorly coded on TriNetX, and therefore we are unable to report on patients who may be living with MetALD (mixed MASLD and moderate alcohol consumption). Given that incretin‐based therapies may be less effective in patients with more advanced disease [13], it is worth highlighting that we are unable to accurately characterise the (histological/biochemical) severity of liver disease (steatosis, fibroinflammation, or fibrosis) in the patients included in this study, as this data is not routinely collected in clinical practice (and therefore available on TriNetX), data restricted to the context of a clinical trial. It is likely that some of our patients may be living with (asymptomatic/pre‐clinical) advanced liver disease, including fibrosis and/or cirrhosis, that is undetected. These patients may have MASLD disease characteristics less responsive to pharmacological treatment. One small (*n* = 14) RCT in patients with MASH treated with liraglutide 1.8 mg found significant reductions in cholesterol‐LDL (−0.7 vs. +0.05 mmol/L; *p* < 0.01), ALT (−54 vs. −4.0 IU/L; *p* < 0.01), and serum leptin, adiponectin, and CCL‐2 (all *p* < 0.05). Metabolic indices also improved with increased suppression of hepatic endogenous glucose production with low‐dose insulin (−9.36 vs. −2.54%; *p* < 0.05) and decreased hepatic de novo lipogenesis in vivo (−1.26 vs. +1.30%; *p* < 0.05). Adipose tissue insulin sensitivity also improved with lower insulin required to half maximally suppress serum non‐esterified fatty acids (−24.9 vs. +54.8 pmol/L; *p* < 0.05) [33]. A further limitation is that cumulative incidence was estimated using the Kaplan–Meier method, which may overestimate risk in the presence of competing risks such as non–MALO death compared to other estimations (i.e., Aalen–Johansen estimator). Unfortunately, the TriNetX platform performs analyses in situ and does not allow users to alter the statistical approach beyond the Kaplan–Meier method. As such, we acknowledge this as a methodological limitation of our study. Importantly, the use of an active comparator design and relatively short follow‐up period (maximum 2 years) is expected to minimise—but not eliminate—the magnitude of potential bias introduced by this limitation.

## 5 Conclusion

Treatment with tirzepatide and, to a lesser extent, semaglutide, in patients with T2D, was associated with a lower incidence of MALO compared with DPP4i after 2 years; largely driven by a reduction in the rates of compensated and decompensated cirrhosis. A reduction in MALO was not demonstrated with the use of liraglutide. This demonstration of hepatoprotection in high‐risk patients with T2D should prompt more robust longer‐term randomised, controlled studies for the use of these drugs for this increasingly prevalent indication.

## Author Contributions

Alex E. Henney was involved in study concept, design, analysis and write up. David R. Riley was involved in study write up, Matthew Anson was involved in study write up, Shazli Azmi was involved in study write up, Uazman Alam was involved in study write up, Daniel J. Cuthbertson was the senior author involved in study concept, design and write up.

## Conflicts of Interest

M.A. receives a fellowship from the Novo Nordisk UK research foundation and JDRF. D.J.C. has received investigator‐initiated grants from Astra Zeneca and Novo Nordisk, support for education from Perspectum with any financial remuneration from pharmaceutical company consultation made to the University of Liverpool and serves as the Topic Advisor for Type 2 Diabetes medications for The National Institute for Health and Care Excellence (NICE), UK. G.H.I. is an employee of TriNetX LLC. U.A. has received honoraria from Procter & Gamble, Viatris, Grunenthal and Sanofi for educational meetings and funding for attendance to an educational meeting from Diiachi Sankyo. U.A. has also received investigator‐led funding by Procter & Gamble and is a council member of the Royal Society of Medicine's Vascular, Lipid & Metabolic Medicine Section. All other authors declare that there are no financial relationships or activities that might bias, or be perceived to bias, their contribution to this manuscript.

## Supporting information


**Data S1:** Supporting Information.

## Data Availability

The data that support the findings of this study are available from TriNetX LLC, https://trinetx.com/, but third‐party restrictions apply to the availability of these data. The data were used under licence for this study with restrictions that do not allow for the data to be redistributed or made publicly available. However, for accredited researchers, the TriNetX data are available for licensing at TriNetX LLC. Data access may require a data sharing agreement and may incur data access fees. Data used in the generation of this paper was collected from the global TriNetX network and local data at LUHFT were not used.
