# Supplementary material for: Target Trial Emulations of GLP‐1 and Dual GLP‐1/GIP Agonists to Reduce Major Adverse Liver Outcomes in Type 2 Diabetes
Source: Liver Int. 2025 Sep 22;45(10):e70367. doi: 10.1111/liv.70367 (PMC12452214; doi:10.1111/liv.70367)
Supplement: Supplementary file 1 — Data S1: Supporting Information. [file LIV-45-0-s001.docx]

**Supplementary Material** Target trial emulations for GLP-1 and dual GLP-1/GIP receptor agonists in reducing major adverse liver outcomes (MALO) in patients with type 2 diabetes

**Table of contents**

**Pages 2-5** Protocol

**Page 6-9** Target trial specification and emulation table (Supplementary Material Table 1)

**Pages 10** Supplementary Material Table 2 (Definitions for all baseline diagnoses, covariates, and outcomes)

**Page 11** Supplementary Material Figure 1 (Density curves produced following propensity score matching)

**Page 12** Supplementary Material Table 3 (Results of sensitivity analysis)

### **Protocol**

#### **1. Title**

- Target trial emulations for GLP-1 and dual GLP-1/GIP receptor agonists in reducing major adverse liver outcomes (MALO) in patients with type 2 diabetes

#### **2. Research Question and Objectives**

- **Research Question**: Can treatment with tirzepatide, semaglutide, and/or liraglutide, compared to DPP4i, reduce the incidence of major adverse liver outcomes in patients with type 2 diabetes?

#### **3. Study Design**

- Target Trial Emulation using retrospective longitudinal data from TriNetX.
- **Study Period**: May 2022 to November 2023 inclusion dates, with 2 years of follow-up from the index event.
- **Population**: Adult patients (≥18 years) living with type 2 diabetes.

#### **4. Eligibility Criteria**

- **Inclusion Criteria**:
  - Medical encounter with HCO in the USA between May 2022 (approval date of tirzepatide for type 2 diabetes by FDA) and November 2023 (approval date of tirzepatide for obesity by FDA)
  - Prescription of tirzepatide, semaglutide, liraglutide, or DPP4i during this time with the date of treatment initiation serving as the index event
  - Diagnosis of type 2 diabetes (defined using: **i)** ICD-10 code E11, and/or **ii)** HbA1c >6.4%) on or before the index event
  - Diagnosis of one or more of the metabolic syndrome components (hypertension, obesity, dyslipidaemia) on or before the index event
- **Exclusion Criteria**:
  - Diagnosis of MALO on or before the index event
  - Contraindications to treatment with tirzepatide, semaglutide, liraglutide or DPP4i (gallstones, pancreatitis, gastroparesis, thyroid cancer) on or before the index event
  - Diagnosis of type 1 diabetes (defined using ICD-10 code)
  - Initiation on another glucose-lowering therapy in the 6 months preceding the initiation of the treatment or reference drug
  - Co-prescription of the treatment or reference drug

#### **5. Interventions**

- **Treatment Group**: Patients receiving one of **i)** tirzepatide; **ii)** semaglutide; **iii)** liraglutide.
- **Comparison Group**: Patients receiving DPP4i.

#### **6. Assignment of Interventions**

- **Definition of Initiation**: Initiation of **i)** tirzepatide; **ii)** semaglutide; **iii)** liraglutide; **iv)** DPP4i, defined as first time the drug is coded on prescription records.
- **Baseline Definition**: The date of initiation of the intervention (or matching initiation date for comparators).

#### **7. Follow-Up**

- **Start of Follow-Up**: Date of intervention/comparator initiation (index event).
- **End of Follow-Up**: Maximum follow-up of 2 years. Patients will be censored if they receive coding for the outcome of interest, at the date of their death or last known fact date, or end of the time-window for analysis.

#### **8. Outcomes**

- **Primary Outcome**: Major adverse liver outcomes (MALO) as a composite outcome
- **Secondary Objectives**: Individual MALO endpoints:
  - Compensated or decompensated cirrhosis (comprising any of the following components: bleeding gastro-oesophageal varices, ascites, hepatorenal syndrome, hepatic encephalopathy),
  - chronic liver failure,
  - hepatocellular carcinoma, or
  - liver transplant

#### **9. Data Sources**

- We aim to explicitly emulate the target trials described using data and built-in analytic functions on the TriNetX Analytics platform. TriNetX (LLC, Cambridge, MA, USA) is a global federated health research network that has access to both inpatient and outpatient electronic medical records from health care organisations internationally; largely secondary, and tertiary care providers in North America and Western Europe. This analysis will be conducted using the US Collaborative Network, which contains data from over 120 million patients (from 69 HCOs) with access to diagnoses, procedures, medications, laboratory values and genomic information worldwide. The built-in analytics within the TriNetX Analytic platform will analyse patient-level data; however, only population-level results will be reported to the research team. TriNetX data are HIPAA (Health Insurance Portability and Accountability Act) de-identified and access to protected health information is not allowed. Therefore, there is no risk for protected health information disclosure, and Institutional Review Board review was not required. Further details on the network have been described by Palchuk, M.B., et al (A global federated real-world data and analytics platform for research. JAMIA Open, 2023. 6(2): p. ooad035).

#### **10. Confounding and Bias Control**

- **Confounding Variables**: Cohorts will be propensity score matched (PSM), in a 1:1 ratio, for **i) sociodemographic variables**: age, sex, ethnicity, smoking, alcohol-use disorder, socioeconomic status (problems relating to education and literacy, employment, housing, and psychosocial circumstances), **ii) comorbidities**: cardiovascular disease (IHD, PVD, HF, CVA), hypertension, dyslipidaemia), **iii) anthropometrics**: body mass index (BMI), and systolic and diastolic blood pressure, **iv) biochemistry**: glomerular filtration rate (GFR), HbA1c, liver enzymes (alanine aminotransferase (ALT), aspartate aminotransferase (AST), and gamma glutamyl transferase (GGT)), clotting (prothrombin time (PT) and activated partial thromboplastin time (APTT), platelets, albumin and triglycerides), and **v) medication** (other blood glucose-lowering therapies): insulin, metformin, sulfonylureas, sodium-glucose cotransporter-2 inhibitors, thiazolidinediones, and other GLP-1 RAs), corticosteroids, diuretics, and aspirin. All biochemical and anthropometric variables used in the cohort creation (i.e., HbA1c and BMI) must have been the most recent recorded value prior to the index event, however we cannot state the exact duration for each patient as we do not have individual level data.
- **Strategies to Address Confounding**:
  - Propensity score matching
  - Emulation of target trials
- **Handling Missing Data**: TriNetX will perform only complete case-analysis.

#### **11. Statistical Analysis Plan**

- **Analysis Population**: Intention-to-treat, per-protocol
- **Primary Analysis**: Survival analysis with an active comparator new-user model
- **Sensitivity Analyses**: We will perform the following sensitivity analyses:
- Head-to-head analyses of the incretin-based therapies (tirzepatide vs. **i)** semaglutide; **ii)** liraglutide; and **iii)** semaglutide vs. liraglutide)

#### **12. Assumptions and Limitations**

- Firstly, these will be real-world data and therefore do not provide randomised or controlled comparisons.
- Secondly, in data extracted from electronic health records in an administrative database, there is potential for a lack of data completeness. This is amplified in the use of open circuit databases like TriNetX where it is possible that a patient may move outside of the HCO and therefore longitudinal data is lost. TriNetX will exclude missing values from any relevant analysis, but it does not provide imputation or any other statistical technique.
- Thirdly, residual bias confounding remains possible despite PSM with potential confounding variables, such as accurate alcohol consumption levels at index, and smoking, both being poorly coded. To address this, we will PSM for liver enzymes at baseline as a biochemical surrogate for alcohol consumption/baseline liver disease severity and will further attempt to reduce the risk of unidentified residual confounding through calculation of E-values as a quantitative bias analysis to assist readers in the interpretation of the strength of our results.

#### **13. Ethical Considerations**

- TriNetX data are HIPAA (Health Insurance Portability and Accountability Act) de-identified and access to protected health information is not allowed. Therefore, there is no risk for protected health information disclosure, and Institutional Review Board review was not required

#### **14. Dissemination Plan**

- Plans to publish findings in peer-reviewed journals and present at conferences.

| **Protocol component** | **Target Trial Specification** | **Target Trial Emulation** |
| --- | --- | --- |
| **Eligibility criteria** | ***Inclusion criteria***   - Adult (≥18 years) patients with type 2 diabetes mellitus based on the World Health Organization classification; diagnosed with T2D before or at baseline - HbA1c between 7.0-9.5% - Stable body weight (+/- 5%) within 3 months of first visit - Eligible for treatment with tirzepatide under prevailing clinical practice guidelines - Evidence of metabolic comorbidity (obesity, hypertension, dyslipidaemia, cardiovascular disease, or HbA1c ≥8.5%)   ***Exclusion criteria***   - Type 1 diabetes - Contraindications to GLP-1/GIP therapy (pancreatitis, medullary thyroid cancer, gallstones, gastroparesis, proliferative diabetic retinopathy or maculopathy) - Initiation of other glucose-lowering therapy within prior 6 months - Pregnancy - eGFR <30 mL/min/1∙73 m^2^ | Same as for Target Trial Specification except for HbA1c upper limit cut off 9.5%. Although some glucose-lowering agent trials mandate patients have a HbA1c between a given range, we did not apply such criteria. Randomised, controlled trials may mandate an upper limit of 10% for HbA1c with insulin then introduced as escalation therapy. However, given that we are using real world data with less concern regarding metabolic safety, we have not prespecified an upper HbA1c limit.  Moreover, patients were excluded if they had a history of MALO before the index event.  We used data from the TriNetX platform between May 2022–Nov 2023. Patients treated with DPP4i mimicked our placebo group from the Target Trial Specification. |
| **Treatment strategies** | - Participants were randomised to receive tirzepatide or placebo. - Those given tirzepatide followed a slow dose escalation regimen fixed at 2∙5 mg-dose increments of tirzepatide every 4 weeks until the maintenance dose was reached. - The maintenance doses of 5, 10, and 15 mg were achieved at 4, 12, and 20 weeks in respective tirzepatide groups. | - Initiation of tirzepatide vs DPP4i (active-comparator, new-user design); with DPP4i acting as the placebo. - Index event is the day of treatment prescription. Intention to treat analysis at assignment. - Three parallel target trials overall in manuscript; this table focuses on tirzepatide vs DPP4i. - Follow-up up to 2 years. |
| **Treatment assignment** | - The central computerized randomisation in SURPASS-1 was done using an interactive web-response system (IWRS). - Participants were allocated (1:1:1:1) to tirzepatide 5 mg, 10 mg, 15 mg, or placebo using a computer-generated random sequence through IWRS. - Randomisation was stratified by country, baseline HbA1c (≤8.5% vs >8.5%), and prior oral glucose lowering therapy use (yes/no). - All participants, investigators, and the sponsor, were blinded; only a very limited support team had access to randomisation data. | - 1:1 propensity-score matching (nearest-neighbour greedy, caliper 0.1 SD). Balance assessed with SMD (<0.1 threshold in practice). - Analyses on matched cohorts. |
| **Outcomes** | ***Primary***   - Change in HbA1c at 40 weeks | ***Primary***   - Incident MALO (compensated, or decompensated (bleeding varices, ascites, hepatorenal syndrome, hepatic encephalopathy), cirrhosis, chronic liver failure, hepatocellular carcinoma or liver transplant).   ***Secondary***   - Individual MALO endpoints, as described above |
| **Follow-up** | Baseline = first injection; follow to 40 weeks + 4-week safety or earlier if outcome/discontinuation; rescue allowed per protocol. | Baseline = day after index prescription; follow to first of outcome, death, loss to follow-up, or 2 years. |
| **Causal contrast** | ICH E9 (R1) treatment-regimen estimand (policy, regardless of discontinuation/rescue) and efficacy estimand (hypothetical, if all adhered and no rescue). | Observational analogue of ITT for tirzepatide vs DPP4i. Sensitivity: per-protocol style in head-to-head incretin analyses requiring ≥6 months adherence and metformin add-on specification. |
| **Analysis** | Graphical testing to control type I error (α=0.05 within estimand); multiple imputation (treatment-regimen estimand); MMRM for efficacy estimand; SAS 9.4. | Kaplan–Meier for cumulative incidence; Cox models for HRs with 95% CIs in matched cohorts; TriNetX built-in analytics (R survival v3.2-3). E-values computed for unmeasured confounding; STROBE followed. |

**Supplementary Material Table 1** Target trial specification (SURPASS-1, Lancet 2021) and emulation (current observational study). The left column describes the protocol components of the randomised controlled trial SURPASS-1. The right column describes how each component was emulated using electronic health records from the TriNetX US Collaborative Network to evaluate tirzepatide *vs.* DPP4 inhibitors for prevention of incident major adverse liver outcomes (MALO).

“Causal contrasts” refer to the policy/intention-to-treat estimand (assignment regardless of adherence or rescue therapy) and the hypothetical/per-protocol estimand (if all patients adhered without rescue).

**Abbreviations:** MALO, major adverse liver outcomes; ITT, intention-to-treat; PSM, propensity score matching; SMD, standardized mean difference; HbA1c, glycated haemoglobin.

| **Diagnosis** | **ICD-10 or CPT code** |
| --- | --- |
| **Inclusion criteria** | |
| Type 2 diabetes | E11 (or HbA1c ≥ 6.5%) |
| Obesity | E66.9 (or BMI >30 kg/m^2^) |
| Hypertension | I10 (or blood pressure ≥140/90 mmHg) |
| Dyslipidaemia | E78 (or triglycerides >150 mg/dL) |
| **Exclusion criteria** | |
| Type 1 diabetes | E10 |
| Gastroparesis | K31.84 |
| Thyroid cancer | C73 |
| Gallstones | K80 |
| Acute pancreatitis | K85 |
| Chronic pancreatitis | K86.1 |
| Major adverse liver outcome | See outcome section |
| **Covariates** | |
| Ischaemic heart disease | I20-I25 |
| Cerebrovascular disease | I60-I69 |
| Peripheral vascular disease | I73 |
| Hypertension | I10 |
| Dyslipidaemia | E78 |
| Nicotine dependence | F17.2 |
| Alcohol-related disorders | F10 |
| Socioeconomic hazards | Z55-Z65 |
| **Outcome** | |
| Compensated liver cirrhosis | K74.6 |
| **Decompensated liver cirrhosis** | |
| Bleeding varices | I85, I86.4 |
| Ascites | R18.8 |
| Hepatorenal syndrome | K76.7 |
| Hepatic encephalopathy | K76.82 |
| Chronic liver failure | K72.1 |
| Hepatocellular carcinoma | C22 |
| Liver transplant | 1007811 |

**Supplementary Material Table 2** Definitions. ICD-10; International Classification of Disease-10, CPT; Current Procedural Terminology.


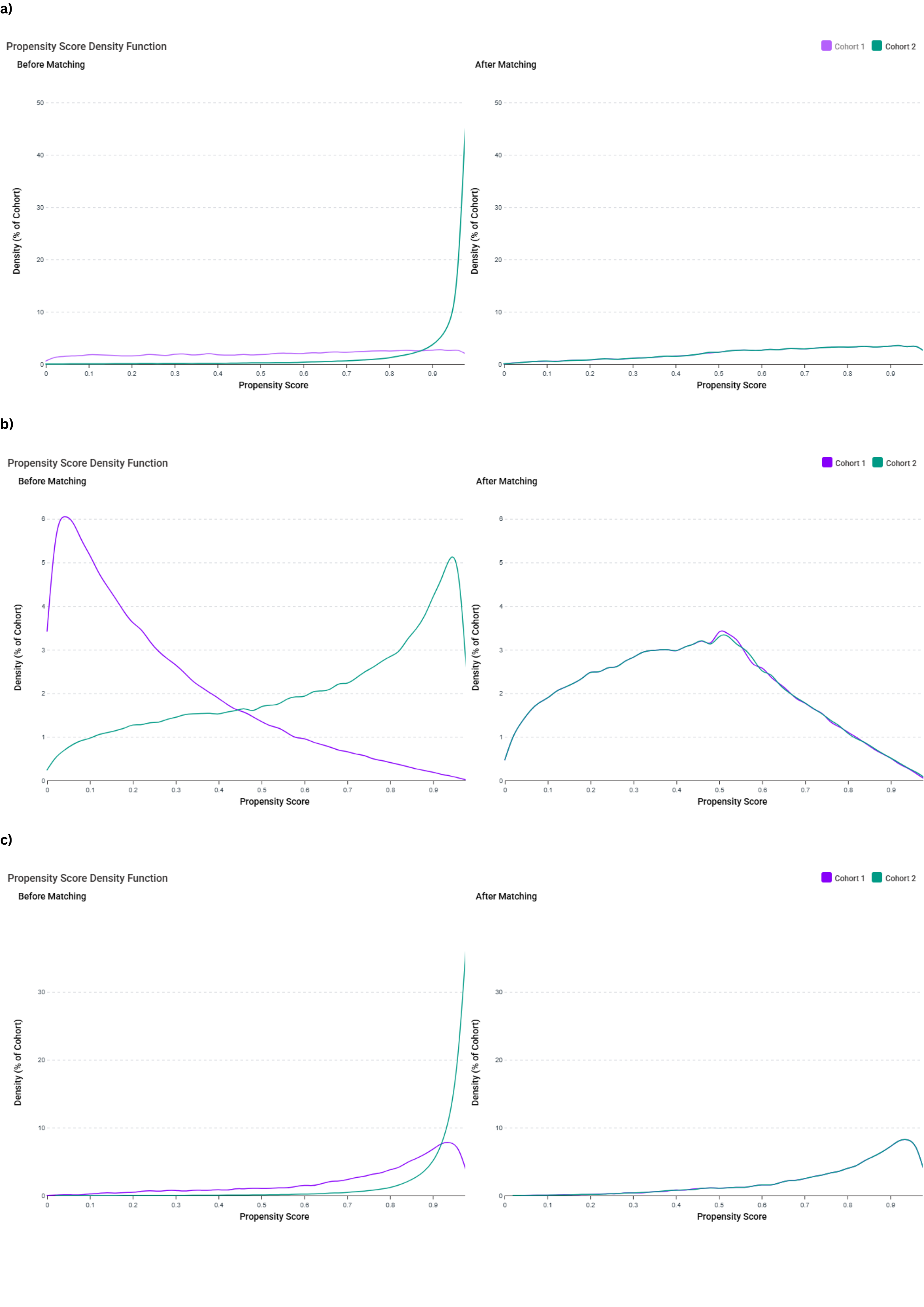


**Supplementary Material Figure 1** Density curves produced following propensity score matching during the following emulated trials: **a)** tirzepatide *vs.* DPP4i, **b)** semaglutide *vs.* DPP4i and **c)** liraglutide *vs.* DPP4i.

| **Sensitivity analysis** | **Hazard ratio [95% confidence interval]** |
| --- | --- |
| ***Tirzepatide vs. DPP4i*** | |
| Geographical location USA only) | **0.57 [0.42, 0.75]** |
| Adjunct to metformin | **0.57 [0.39, 0.83]** |
| Treatment adherence | **0.57 [0.40, 0.80]** |
| ***Semaglutide vs. DPP4i*** | |
| Geographical location USA only) | **0.74 [0.66, 0.83]** |
| Adjunct to metformin | **0.75 [0.65, 0.86]** |
| Treatment adherence | **0.75 [0.65, 0.86]** |
| ***Liraglutide vs. DPP4i*** | |
| Geographical location USA only) | 1.08 [0.82, 1.42] |
| Adjunct to metformin | 1.05 [0.72, 1.51] |
| Treatment adherence | 0.91 [0.66, 1.24] |

**Supplementary Material Table 3** Sensitivity analysis results according to geographical location, co-existing metformin therapy and treatment adherence, with results in bold representing statistical significance.
